# Antidepressant-like effects of auraptenol in mice

**DOI:** 10.1038/srep04433

**Published:** 2014-03-24

**Authors:** Xiaosu Gu, Yong Zhou, Xiaomei Wu, Fen Wang, Cai-Yi Zhang, Chenchen Du, Lihua Shen, Xiang Chen, Jiansheng Shi, Chunfeng Liu, Kaifu Ke

**Affiliations:** 1 The Department of Neurology, Second Affiliated Hospital of Soochow University, Suzhou, Jiangsu, 215000 China; 2 Institute of Neuroscience; Soochow University; Suzhou, Jiangsu, 215123 China; 3 Department of Neurology, Affiliated Hospital of Nantong University, Nantong, Jiangsu, 226001 China; 4 The Department of Neurology, The First People's Hospital of Nantong, Nantong, Jiangsu, 226001 China; 5 Department of Neuropharmacology, Institute of Nautical Medicine, Nantong University, Nantong, Jiangsu, 226001 China

## Abstract

Depression is a major psychiatric disorder affecting nearly 21% of the world population and imposes a substantial health burden on society. Current available antidepressants are not adequate to meet the clinical needs. Here we report that auraptenol, an active component of the traditional Chinese medicine, angelicae dahuricae radix, had antidepressant-like effects in mice models of depression. In mouse forced swimming test and tail suspension test, two validated models of depression, auraptenol dose-dependently decreased the immobility duration within the dose range of 0.05–0.4 mg/kg. In addition, the antidepressant-like effects of auraptenol was significantly averted by a selective serotonin 5-HT_1A_ receptor antagonist WAY100635 (1 mg/kg). These doses that affected the immobile response did not affect locomotor activity. In summary, this study for the first time identified an active component from the herbal medicine angelicae dahuricae radix that possesses robust antidepressant-like efficacy in mice. These data support further exploration for the possibility of developing auraptenol as a novel antidepressant agent in the treatment of major depression disorders.

Depression is a major psychiatric disorder affecting nearly 21% of the world population and imposes a substantial health burden on society^1^,^2^. There are three main kinds of classical antidepressants in clinical practice, including tricyclic antidepressants, selective serotonin reuptake inhibitors (SSRIs) and monoamine oxidase inhibitors (MAOIs). Most of these drugs, however, have undesirable side effects and their mechanisms of action have not been satisfactorily resolved. A growing number of herbal medicines are being introduced into psychiatric practice, many of which have comparable efficacy to prescription medications with lower side effects. This makes herbal therapies as desirable alternative treatment for severe depression^3^.

Angelicae dahuricae radix is a perennial plant that grows naturally in broad areas of China. Angelicae dahuricae radix has a strong scent and its leaves are used to make incense. In addition, the roots of angelicae dahuricae radix (also known as Bai Zhi) are used in traditional Chinese medicine to treat harmful external influences on the skin, such as cold, heat, dampness and dryness^4^. Modern pharmacological studies on angelicae dahuricae radix have reported that crude extracts of angelicae dahuricae radix possesses anti-inflammatory, analgesic and antipyretic actions and acute toxicity as a guideline for clinic application^4^. Essential oil of angelicae dahuricae radix has analgesic effect in rat models of pain, and the antinociceptive effects have been linked to the facilitated release of endogenous opioids such as beta-endorphin^5^. More importantly, angelicae dahuricae radix has been used clinically to treat depressive symptoms^6^. However, the crude extract and essential oil of angelicae dahuricae radix include multiple potentially active chemical compounds and the active ingredient(s) of angelicae dahuricae radix that are responsible for its antidepressant-like activity are currently unknown. Recent phytochemical research has purified and identified several active coumarin components of angelicae dahuricae radix^7^, and further pharmacological studies are needed to identify the active coumarin component underlying the antidepressant-like actions of angelicae dahuricae radix.

This study reported the potent antidepressant-like effects of an active coumarin component of angelicae dahuricae radix, auraptenol (8-(2-hydroxy-3-methylbut-3-en-1-yl)-7-methoxy-2*H*-chromen-2-one, in mice models of depression. Receptor mechanism underlying the antidepressant-like effects of auraptenol was also studied.

## Results

**Figure 1:**
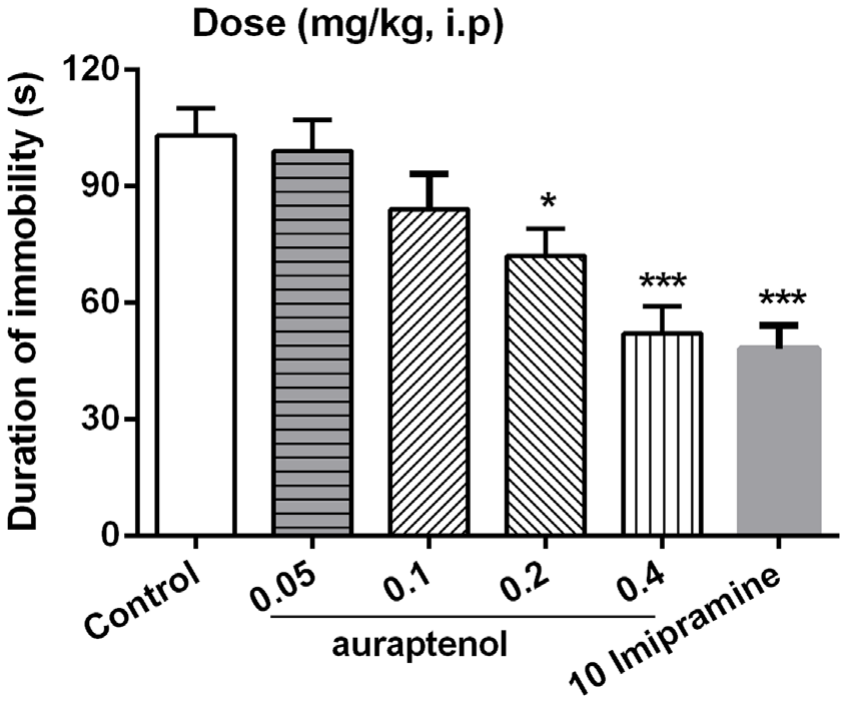
The effects of auraptenol on the duration of immobility in the forced swimming test. Mice were administered vehicle, auraptenol (0.05, 0.1, 0.2, 0.4 mg/kg) or imipramine (10 mg/kg). Values were the mean ± S.E.M. with 8 mice in each group. * P < 0.05, *** P < 0.001 vs. the vehicle control group.

As shown in Figure 1, the potential antidepressant-like effects of auraptenol were evaluated in mouse forced swimming test. Auraptenol showed a dose-dependent reduction of the duration of immobility after acute drug administration, leading to a maximum of 46.3% immobility reduction at a dose of 0.4 mg/kg. The classical antidepressant agent imipramine (10 mg/kg, i.p.) also significantly decreased the immobility time that reached 53.4% reduction of the immobility time. One way ANOVA revealed a significant effect of auraptenol dose ([F (4, 35) = 12.43, P < 0.001 vs. control group]. Post hoc analyses indicated that 0.2 and 0.4 mg/kg auraptenol significantly decreased the immobility time in mice (Fig. 1).

**Figure 2:**
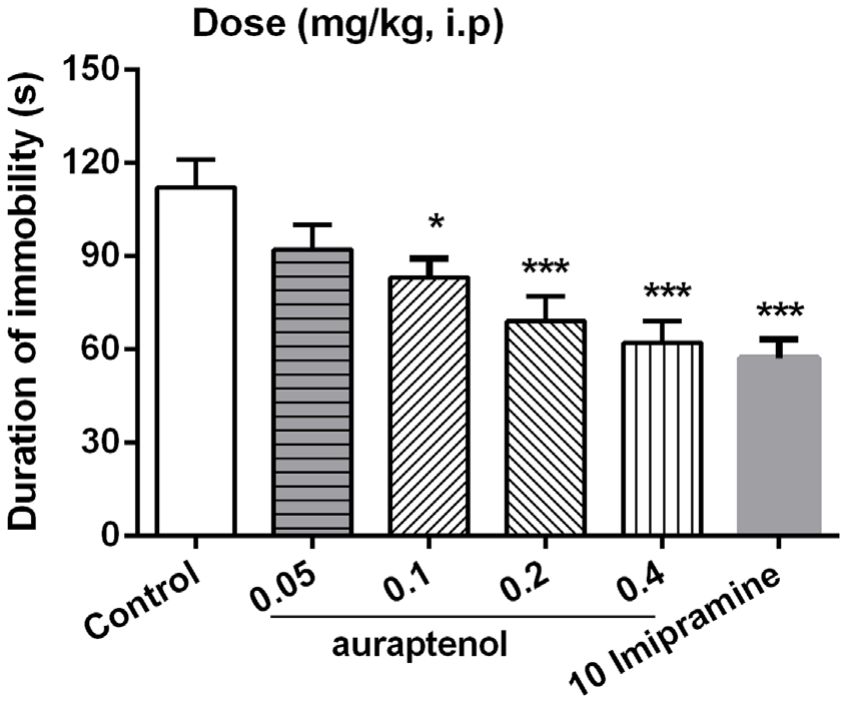
The effects of auraptenol on the duration of immobility in the tail suspension test. Mice were administered vehicle, auraptenol (0.05, 0.1, 0.2, 0.4 mg/kg) or imipramine (10 mg/kg). Values were the mean ± S.E.M. with 8 mice in each group. * P < 0.05, *** P < 0.001 vs. the vehicle control group.

Figure 2 showed the potential antidepressant-like effects of auraptenol in mouse tail suspension test. Auraptenol showed a dose-dependent reduction of the duration of immobility after acute drug administration, leading to a maximum of 44.7% immobility reduction at a dose of 0.4 mg/kg. The classical antidepressant agent imipramine (10 mg/kg, i.p.) also significantly decreased the immobility time that reached 49.1% reduction of the immobility time. One way ANOVA revealed a significant effect of auraptenol dose ([F (4, 35) = 21.46, P < 0.001 vs. control group]. Post hoc analyses indicated that 0.1, 0.2 and 0.4 mg/kg auraptenol significantly decreased the immobility time in mice (Fig. 2).

**Figure 3:**
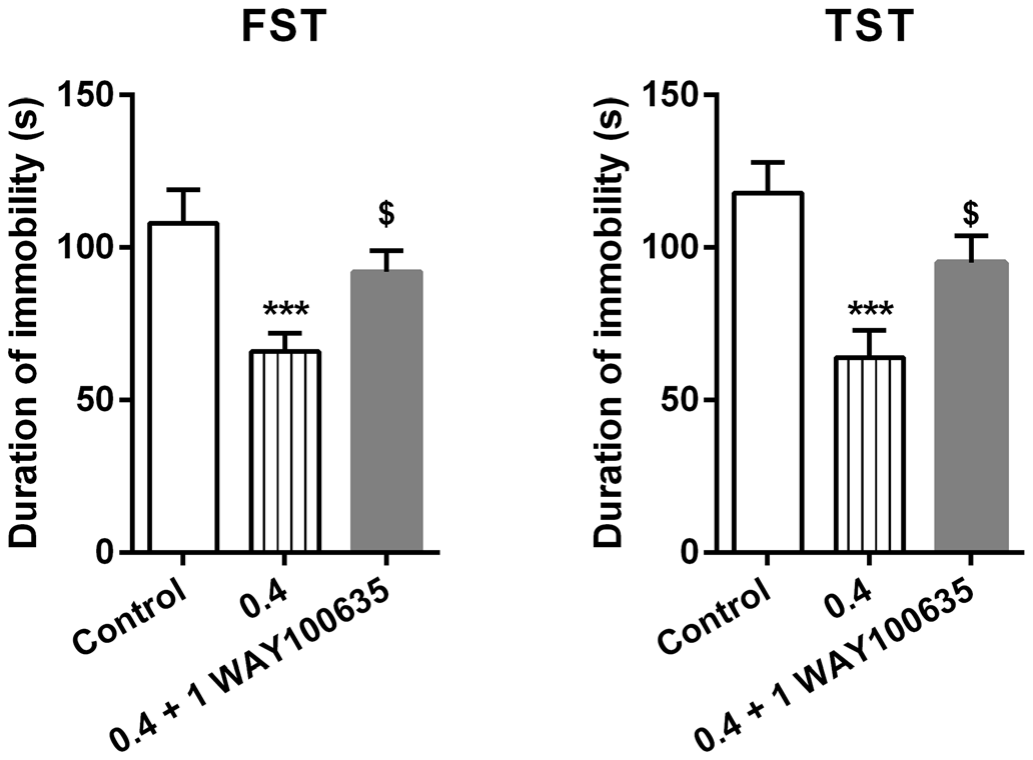
Effect of the 5-HT_1A_ receptor antagonist WAY100635 on the antidepressant-like effects of auraptenol in forced swimming test (FST) and tail suspension test (TST) (n = 8 per group). *** P < 0.001 as compared to control group; ^$^ P < 0.05 as compared to 0.4 mg/kg auraptenol group.

In order to examine the potential receptor mechanism of auraptenol in both mouse models of depression, the selective 5-HT_1A_ receptor antagonist WAY100635 was studied as a pretreatment with auraptenol in the behavioral assays. In both assays, a dose of 1 mg/kg WAY100635 pretreatment significantly blocked the effects of auraptenol (F [2, 21] = 9.42, P < 0.05 in forced swimming test; F [2, 21] = 11.04, P < 0.01) (Fig. 3).

**Figure 4:**
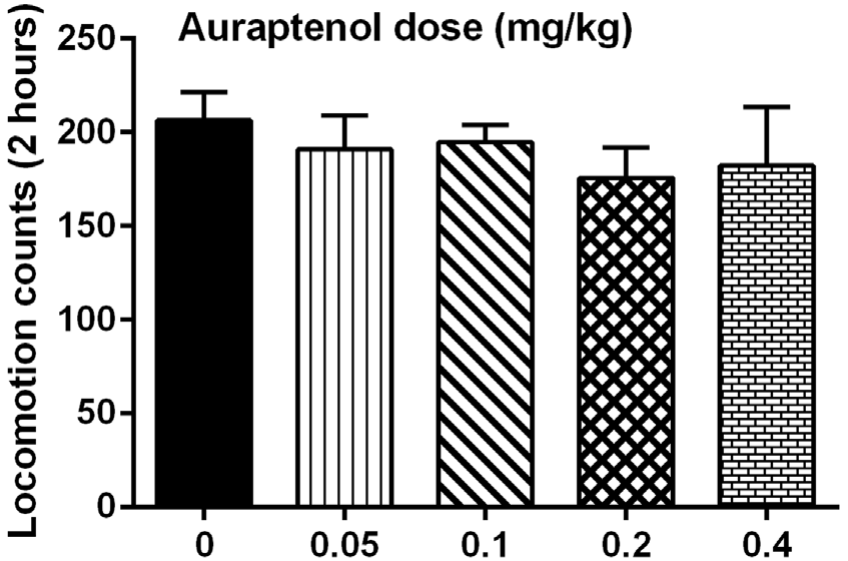
Effects of auraptenol on the general locomotor activity in mice (n = 8 per group).

The potential effect of auraptenol treatment on the general locomotor activity in naïve mice was examined with different doses of auraptenol (Fig. 4). It was found that auraptenol had no significant effect in decreasing the general locomotor activity in mice across the dose range studied. One-way ANOVA found no significant effect on auraptenol dose (F [4, 35] = 0.48, P > 0.05). Post hoc analysis revealed that none of the auraptenol doses significantly altered the locomotor activity (Fig. 4).

## Discussion

Here we reported that an active component from the plant, angelicae dahuricae radix, produced potent anti-depressant-like effects in two well-validated mouse models of depression, forced swimming test and tail suspension test. Preliminary pharmacological mechanism study also revealed a prominent 5-HT_1A_ receptor mediated effect. Finally, we tested the behavioral specificity of the observed effects by examining the effects of auraptenol on the general locomotor activity in mice and found no significant effect of auraptenol on the mice's overall locomotion. Although angelicae dahuricae radix has been used in traditional medicine for centuries to treat various malaises, contemporary pharmacological studies just begin to discern the active components in this plant that are responsible for its various therapeutic actions. This study represents the first that identified the potential antidepressant effects of auraptenol in widely used mouse models of depression. These encouraging results call for more detailed pharmacological studies of this new compound for its potential to treat depression and other mental disorders alike.

Depression is the outcome of an eventual inability to cope with a stream of dissimilar unpleasant stimuli imposed by the environment. Animal models are widely used in pre-clinical antidepressant evaluation and to provide insights into the neuropathology of depression^12^. The forced swimming and tail suspension tests are the most widely used tools for inducing behavioral deficits which can subsequently be reversed by antidepressant treatments. There is a significant correlation between clinical potency and effectiveness of antidepressants in both models^13^. The present study demonstrated that auraptenol at systemic doses of 0.2 mg/kg or higher exerted a significant effect to reduce the immobility time after acute administration both in the tail suspension and forced swimming tests in mice. Acute treatment with the classical antidepressant imipramine (10 mg/kg, i.p.) also significantly reduced the immobility time. The antidepressant-like effects of auraptenol were largely comparable to those found with imipramine (10 mg/kg, i.p.). Furthermore, we did not observe any evidence indicating a change in locomotor activity in mice at the doses that significantly improved antidepressant performance. Thus, the antidepressant-like effect of auraptenol is unlikely to be due to an increase in locomotor activity. These results clearly demonstrated for the first time, the antidepressant-like efficacy of auraptenol in the mouse models of depression.

Serotonin 5-HT_1A_ receptors have been shown to involve the pathophysiology of depression and drugs activating 5-HT_1A_ receptors possess robust antidepressant-like effects in various rodent models of depression^14^. For example, 5-HT_1A_ receptor agonists decrease the duration of immobility in a rat forced swimming test and the effectiveness of the ligands are positively correlated with their efficacy for activating 5-HT_1A_ receptors^15^. In this study, the antidepressant-like effects of auraptenol were significantly blocked by a selective 5-HT_1A_ receptor antagonist, WAY100635, in both mouse models of depression. This results strongly suggest the dominant role of 5-HT_1A_ receptors in the observed antidepressant-like effects of auraptenol. This result is significant because auraptenol is a compound that exists in the nature and could provide a broad range of utilities related to 5-HT_1A_ receptor agonism. It should be noted that the fact that the effect of auraptenol is blocked by 5-HT_1A_ receptor antagonists does not automatically translate to 5-HT_1A_ receptor agonism. A secondary mechanism is also possible in that auraptenol works on a certain target which in turns functionally activates 5-HT_1A_ receptors. We know of no studies that directly measure the receptor binding of auraptenol and future studies that utilize receptor binding technique will directly address this issue.

In summary, this study for the first time demonstrated that auraptenol has a potent antidepressant-like effect in two mouse models of depression, without apparent adverse effects (motor alteration). The finding that the antidepressant-like effect of auraptenol was primarily mediated by activation of 5-HT_1A_ receptors is consistent with a large body of literature that 5-HT_1A_ receptors are critically involved in the etiology and pathophysiology of depression. Thus, these data may suggest another potential antidepressant agent that works through 5-HT_1A_ receptors and this hypothesis is worth testing in more details in future studies.

## Methods

### Animals

Male C57BL/6 mice weighing 16–22 g (Weitong Lihua, Beijing, China) were acclimated to the temperature, humidity and lighting (12 h light/dark cycle, lights on at 7:00 AM) controlled vivarium and housed in groups of four for at least one week before behavioral studies began. The animals had free access to dietary food and water except during the test sessions. All animal experimental protocols were approved by the Soochow University Committee on Animal Care and Use. Animals were maintained and experiments were conducted in accordance with the *Guide for the Care and Use of Laboratory Animals* (8^th^ edition, Institute of Laboratory Animal Resources on Life Sciences, National Research Council, National Academy of Sciences, Washington DC). Animals were used only in one experiment.

### Drugs

Imipramine hydrochloride and WAY100635 maleate salt were purchased from Sigma-Aldrich (St. Louis, MO, USA) and were dissolved in physiological saline. Auraptenol was purchased from Shanghai Lei Yun Shang Pharmaceutical Co. (>95% purity, Shanghai, China) and was dissolved in saline with one drop of acetic acid. Except otherwise noted, all injections were given intraperitoneally in a volume of 1 ml/100 g of body weight.

### Behavioral procedures

#### Forced swimming test

The forced swimming test employed was similar to that described elsewhere^8^,^9^ with minor modification. Briefly, mice had a swimming-stress session for 15 min (pre-test), 24 h before being individually placed in glass cylinders (height: 25 cm; diameter: 10 cm; containing 10 cm of water at 24 ± 1°C) for 6 min (test). A camera was mounted beside the forced swimming cylinder and all the test events were recorded. Two experienced observers independently scored the behavior blindly and an average of the two scores were used as a final score. A mouse was judged to be immobile when it ceased struggling and remained floating motionless in the water, making only small movements necessary to keep its head above water. The duration of immobility was recorded during the last 4 min of the 6-min testing period.

#### Tail suspension test

The tail suspension test was based on the method of Steru^10^. Animals were suspended 50 cm above the floor by means of an adhesive tape, placed approximately 1 cm from the tip of the tail. The time during which mice remained immobile was quantified during a test period of 6 min. Mice were considered immobile only when they hung passively and completely motionless. A camera was mounted facing the tail suspension test arena and all the test events were recorded. Two experienced observers independently scored the behavior blindly and an average of the two scores were used as a final score.

#### Locomotor activity

The locomotor activity of naïve mice treated with vehicle or auraptenol was measured automatically with a Small Animal Locomotion Recording Apparatus (Shandong Academy of Medical Sciences, China)^11^, which consisted of six acrylic boxes and in each box there was one pyroelectric infrared sensor 4 cm above the floor. The sensor could detect the movements of the mice through infrared radiation. The apparatus recorded only gross movements of the mice, whereas small movements such as gnawing or grooming could not be differentiated and recorded. Immediately after drug or vehicle treatment, the mice were put into the boxes and their general locomotor activity was measured for a period of 2 hours.

### Data analyses

Results were expressed as the mean ± standard error of the mean (S.E.M.). All data were analyzed statistically using one-way analysis of variance (ANOVA), followed by a post hoc Dunnett's test. Differences with P < 0.05 were considered statistically significant.

## Author Contributions

C.L., J.S. and K.K. designed the experiments; X.G., Y.Z., X.W., F.W. and C.Z. conducted the experiments; C.L., C.D. and K.K. wrote the main manuscript text; L.S. and X.C. conducted the statistical analyses and prepared the figures. All authors reviewed and approved the manuscript.
